# Is Appendiceal Diverticulitis Mimicking Acute Appendicitis?

**DOI:** 10.7759/cureus.51214

**Published:** 2023-12-28

**Authors:** Mohammed N AlAli, Nardeen I Alsweed, Ameen Alshehri, Mohamed S Essa, Saud K Aldeghaither, Mohammad A Meaigel, Muath Alrashed, Abdulbaset M Al-Shoaibi, Sadiq M Amer, Mohammed H Alsubaih

**Affiliations:** 1 Department of Surgery, Prince Mohammed Bin Abdulaziz Hospital, Ministry of Health (MOH), Riyadh, SAU; 2 Department of Clinical Surgery, College of Medicine, Princess Nourah Bint Abdulrahman University, Riyadh, SAU; 3 Department of Surgery/Acute Care and Trauma Surgery, King Khalid University Hospital, King Saud University, Riyadh, SAU; 4 Department of Radiology, Prince Mohammed Bin Abdulaziz Hospital, Ministry of Health (MOH), Riyadh, SAU; 5 Department of Pathology, Prince Mohammed Bin Abdulaziz Hospital, Ministry of Health (MOH), Riyadh, SAU

**Keywords:** acute abdomen, appendicitis, appendectomy, appendicular diverticulitis, appendicular diverticulosis

## Abstract

Appendicular diverticulitis (AD) is a rare entity characterized by the inflammation of the arising diverticulum of the appendix. It has been reported to carry a high risk of perioperative complications, such as bleeding and perforation. Furthermore, multiple articles have highlighted the importance of diagnosing AD early due to its strong association with malignancies. Limited published cases concerning AD in our country and globally are available in the literature. Hence, we present in this article a case series of five exciting cases of incidental findings of AD that were initially diagnosed as acute appendicitis based on clinical evaluation and imaging findings. In our series, we performed a retrograde evaluation of the computed tomography scans of all five cases that showed diverticula. In conclusion, histopathological evaluation remains the method of choice to reach the definitive diagnosis; however, it is essential to highlight the relevance of imaging in diagnosing AD preoperatively in the early stages to reduce morbidity and mortality.

## Introduction

Appendicular diverticulitis (AD) is a rare entity of the appendix, first described in 1893 [1]. The incidence of AD ranges between 0.014% and 1.9% and is up to 2.1% among appendectomy cases [1-3]. It is categorized into congenital and acquired diverticula (more common) [2]. Male gender, age > 30 years, chronic appendicitis, cystic fibrosis, and Hirschsprung’s disease are known risk factors for AD [4]. AD carries a higher risk of bleeding, perforation, and appendiceal neoplasms, with a higher rate of mortality than acute appendicitis (AA) [4-6]. In addition, the mean operation duration and postoperative hospital stay were longer than in AA [6]. Furthermore, multiple published studies have highlighted the importance of diagnosing AD earlier due to its strong association with malignancies, such as carcinoid tumors and mucinous adenoma [7]. It is challenging to differentiate it clinically from AA as they share a similar clinical presentation and radiological features; therefore, most AD cases are diagnosed intraoperatively or during histopathological evaluation [8-10].

A limited number of cases concerning AD are available in the literature (approximately 155 cases), and only three cases were reported in our country, and a few cases in the Arab Gulf region [4-7]. Therefore, we present a case series of five interesting incidental findings of AD that were initially diagnosed as AA based on intraoperative and histopathological findings.

## Case presentation

Case 1

**Figure 1:**
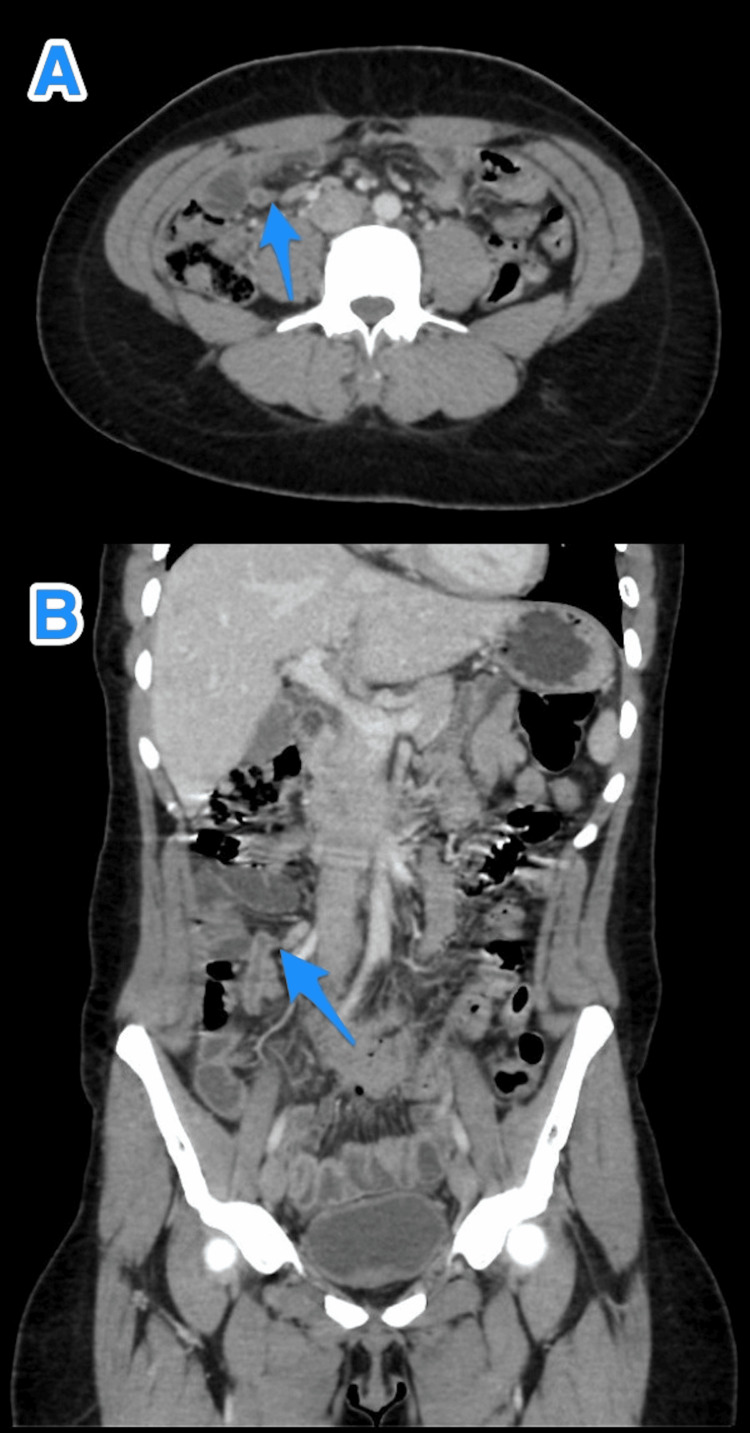
A multiview computed tomography abdominal scan (A and B) shows a small diverticulum close to the tip of an inflamed appendix (arrows).

**Figure 2:**
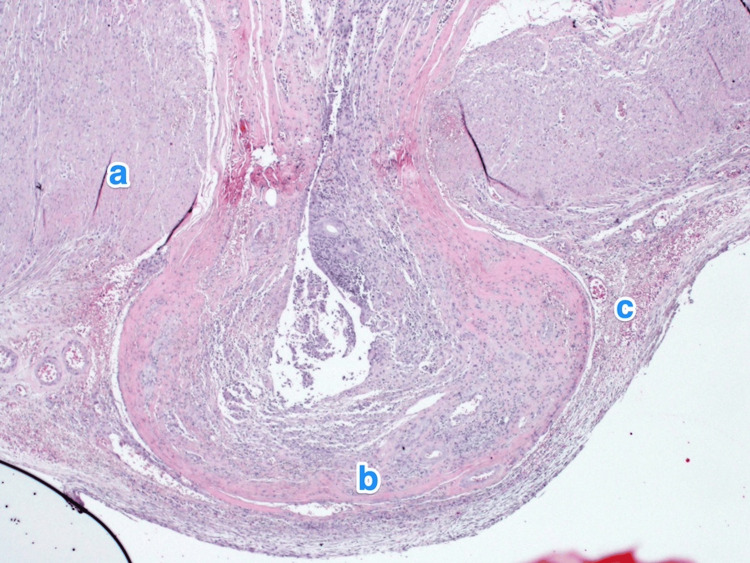
Histopathology of the appendix. A light microscopy photograph of the appendix shows multiple small outpouchings of the mucosa and submucosa through the muscular layer (a) of the appendicular tip and lateral side. The mucosa of the diverticula is lined by columnar epithelium, showing ulceration and active inflammation (b) (diverticulitis). The submucosa also shows mild fibrosis and chronic inflammatory infiltrates (c). Hematoxylin and eosin stain, 2x.

A 32-year-old female with no prior medical illnesses but with a history of unspecified gynecological surgery presented to our institution with a typical appendicitis history and a two-day history of shifted right lower quadrant (RLQ) abdominal pain associated with nausea, vomiting, anorexia, and fever. She had no prior history of similar episodes, constitutional symptoms, changes in bowel motion, gynecological symptoms, or family or personal history of malignancy with a negative systematic review. The patient looked well upon examination, and vital signs and hemodynamics were within normal ranges. The abdomen was not distended with tenderness and rebound tenderness in the RLQ; otherwise, it was unremarkable (including negative other appendicitis signs). Further, the patient had a normal white blood cell (WBC) count (9.93 × 109/L) (reference range: 4.5-11.0 x 109/L). A preoperative abdominal computed tomography (CT) scan revealed acute uncomplicated appendicitis, while the postoperative review showed a small diverticulum close to the tip of an inflamed appendix (Figure 1). However, the patient underwent a laparoscopic appendectomy with an uneventful postoperative course. The intraoperative findings showed an inflamed appendix with a nodule over the serosal surface. The patient was discharged on postoperative day one in good condition. Acute suppurative AD without an appendicolith or malignant cells was observed on the histopathological examination (Figure 2). Further, the patient was doing well on regular follow-ups (two and six weeks postoperatively).

Case 2

A 21-year-old Saudi male with unremarkable past medical and surgical history presented to the emergency department (ED) with a three-day history of RLQ abdominal pain, which increased in intensity on the day of presentation. Initially, the pain was in the periumbilical region, then shifted to the RLQ associated with anorexia. He had no previous history of similar episodes, constitutional symptoms, or personal and family history of malignancy, and the systemic review was unremarkable. Upon physical examination, he looked fine, with normal vitals, and hemodynamically stable. The abdominal examination revealed a non-distended abdomen with RLQ tenderness and negative rebound tenderness. Other appendicitis signs were all negative. WBC count was within the normal range (6.35 × 109/L) (reference range: 4.5-11.0 x 109/L).

**Figure 3:**
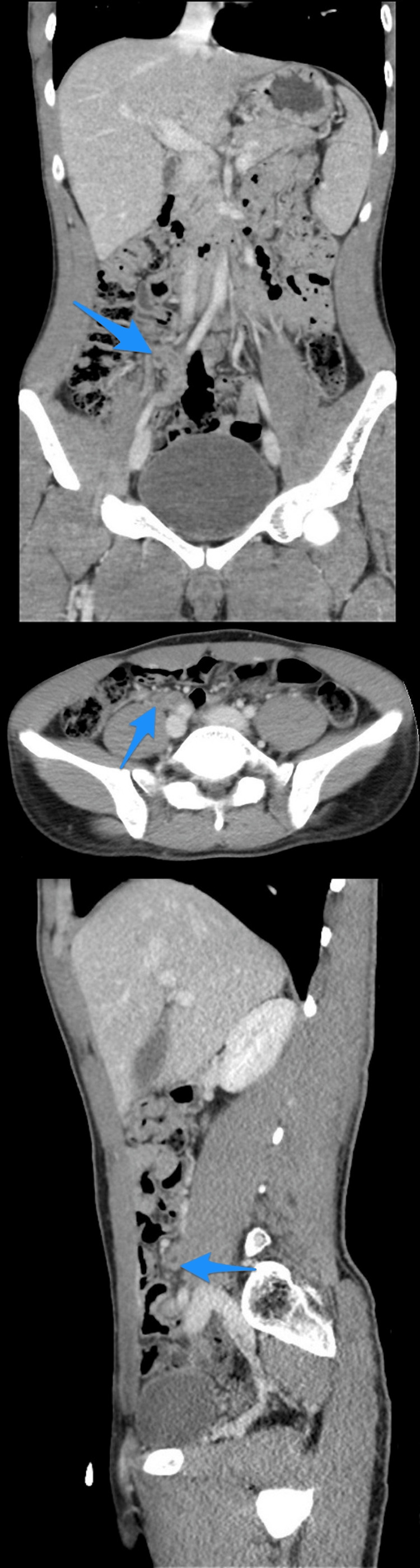
A multiview computed tomography abdominal scan (A-C) shows a small diverticulum close to the tip of an inflamed appendix (arrows).

**Figure 4:**
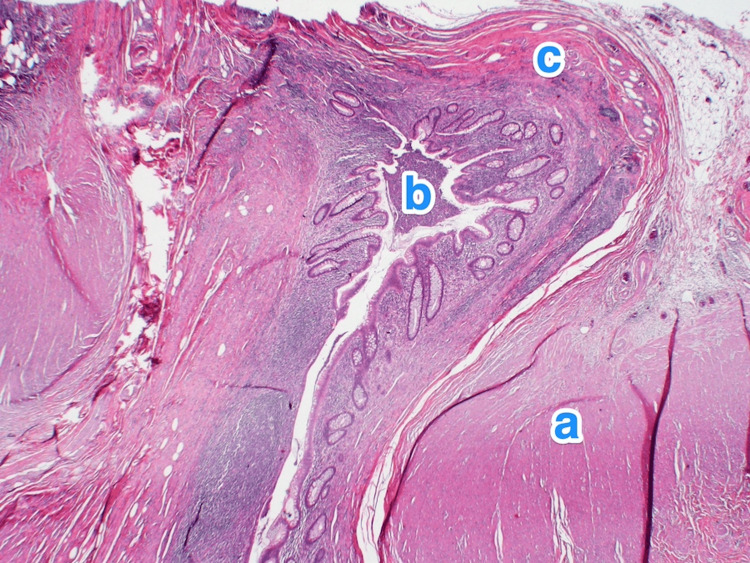
Histopathology of the appendix. A light microscopy photograph of the appendix shows multiple small outpouchings of the mucosa and submucosa through the muscular layer (a) of the appendicular tip and lateral side. The mucosa of the diverticula is lined by columnar epithelium, showing active inflammation (b) (diverticulitis) and luminal pus. The submucosa also shows mild fibrosis and chronic inflammatory infiltrates (c). Hematoxylin and eosin stain, 2x.

A preoperative abdominal CT scan showed uncomplicated AA. In contrast, a retrograde assessment of the radiological images showed a small diverticulum close to the tip of an inflamed appendix (Figure 3). The patient underwent an open appendectomy on the same day of presentation with no identifiable complications. The intraoperative assessment revealed an inflamed appendix with no diverticulosis. The postoperative course was uneventful, and the patient was discharged on the next day in good health. Acute suppurative AD with no evidence of malignancy was observed in the histopathological report (Figure 4). During the follow-up in the clinic two weeks postoperatively, the patient was doing fine with no active complaints.

Case 3

A 25-year-old male with no significant past medical and surgical history presented to our institution with a complaint of RLQ abdominal pain for four days. On the day of the presentation, the non-radiating pain increased suddenly in its severity; it was the first time the patient encountered this pain. The pain was associated with nausea, vomiting, cough, and rhinorrhea. The patient denied having constitutional symptoms, and the systemic review was unremarkable. He had no history of previous hospital admissions and malignancy. The patient generally looked fine and not distressed, and hemodynamics were within normal ranges. The abdomen was soft, not distended, with a right iliac fossa tenderness and positive rebound tenderness; otherwise, it was unremarkable. WBC count was within the normal limits (9.8 × 109/L) (reference range: 4.5-11.0 x 109/L).

**Figure 5:**
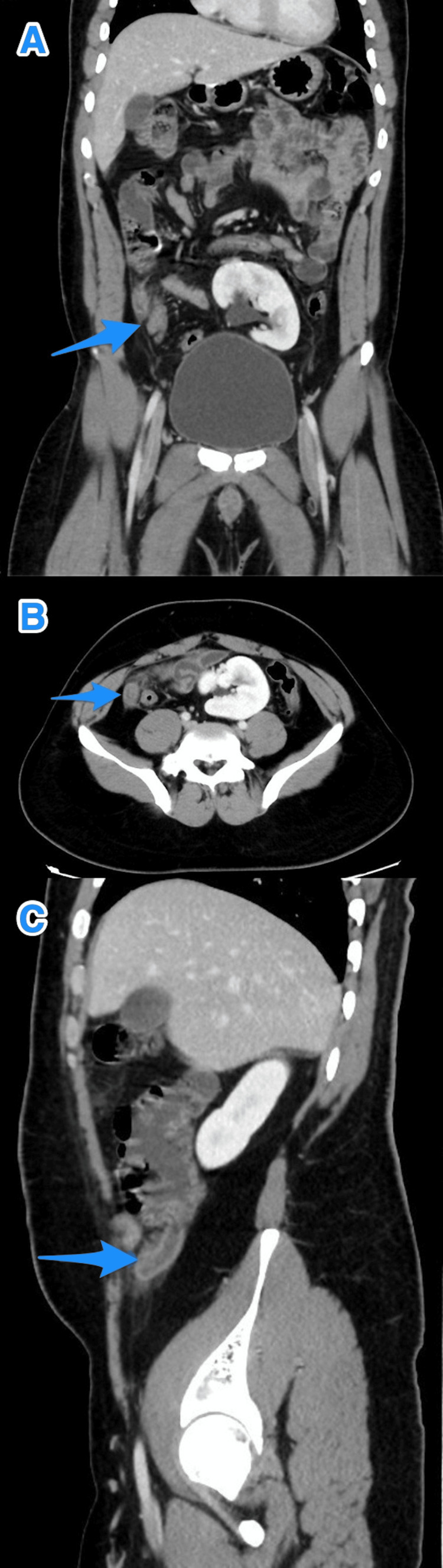
A multiview computed tomography abdomen scan (A-C) shows a small diverticulum close to the tip of an inflamed appendix (arrows).

**Figure 6:**
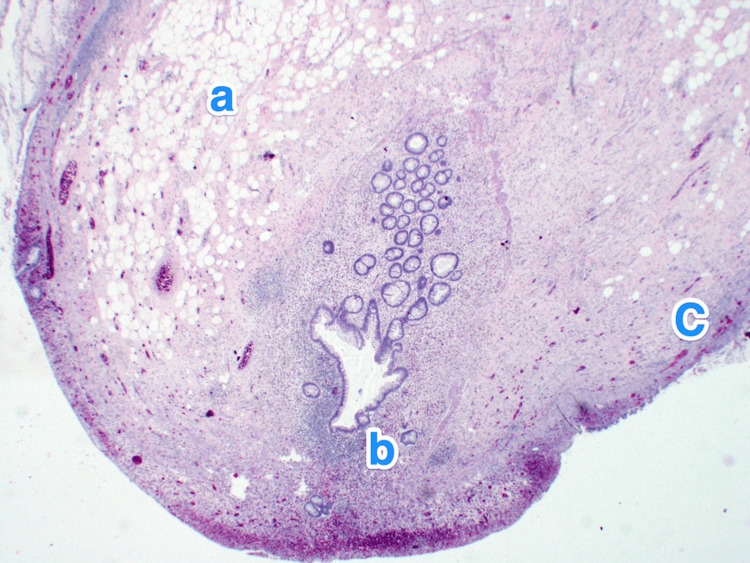
Histopathology of the appendix. A light microscopy photograph of the appendix shows multiple small outpouchings of the mucosa and submucosa through the muscular layer of the appendicular tip and lateral side into subserosal space (a). The mucosa of the diverticula is lined by columnar epithelium, showing active inflammation (b) (diverticulitis). The submucosa also shows mild fibrosis and chronic inflammatory infiltrates (c). Hematoxylin and eosin stain, 2x.

A preoperative abdominal CT scan was suggestive of uncomplicated AA, while postoperative assessment showed a small diverticulum close to the tip of an inflamed appendix (Figure 5). The patient underwent open appendectomy, and the procedure was done with no complications and revealed a suppurative appendix with no evidence of diverticula or perforation. The hospital course was uneventful, and the patient was discharged with no active complaint. AD with peri-appendicitis without identified malignant cells was observed in the histopathological study (Figure 6). At two weeks postoperative follow-up in our clinic, the patient was doing well with no active complaints.

Case 4

A 26-year-old female with unremarkable past medical and surgical history presented to the primary healthcare office with RLQ abdominal and flank pain for two weeks. A CT showed features of AA. Thus, the patient was transferred to the ED for further assessment. The pain was intermittent, lasting for hours; the last attack was on the day of presentation and was associated with dysuria. The patient denied constitutional symptoms, changes in bowel motion, and gynecological symptoms. In addition, there were no previous hospital admissions, no history of trauma, and she was not on any medications. Upon examination, she was looking well, afebrile, and vitally stable. Abdominal examination revealed a non-distended abdomen with mild right iliac fossa tenderness and negative rebound tenderness. Otherwise, the examination was unremarkable. WBC count was within normal limits (6.1 × 109/L) (reference range: 4.5-11.0 x 109/L).

**Figure 7:**
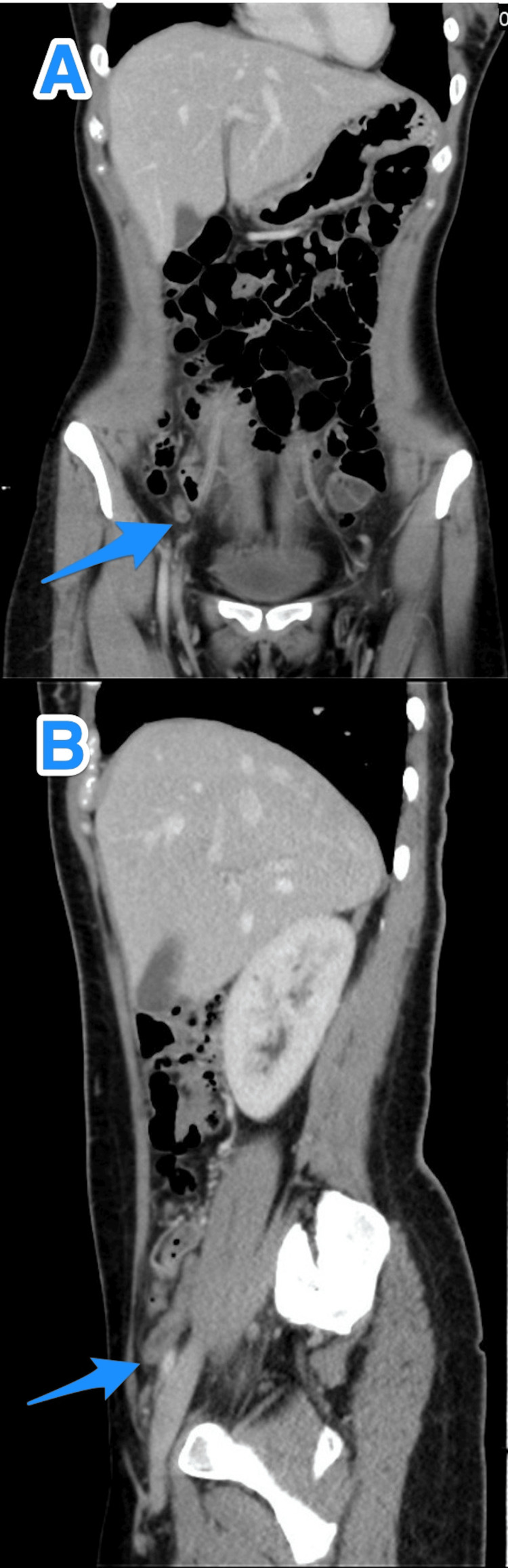
A multiview computed tomography abdomen scan (A and B) shows a small diverticulum close to the tip of an inflamed appendix (arrows).

**Figure 8:**
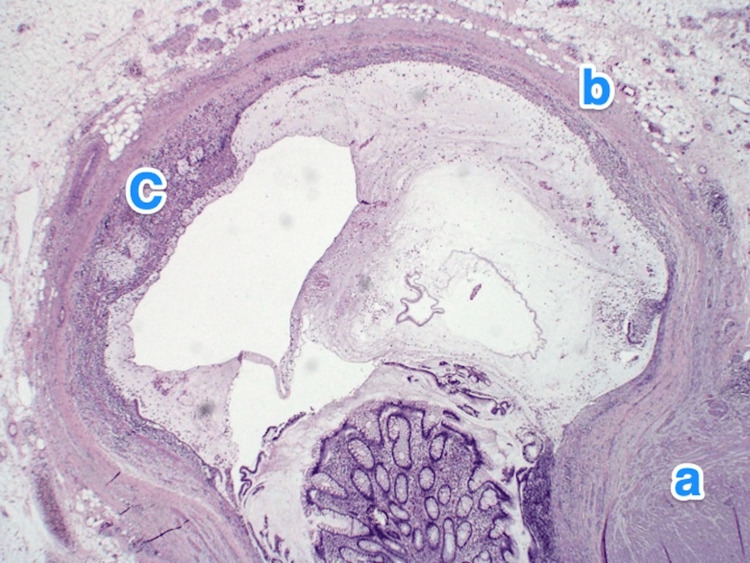
Histopathology of the appendix. A light microscopy photograph of the appendix shows multiple small outpouchings of the mucosa and submucosa through the muscular layer (a) of the appendicular tip and lateral side. The mucosa of the diverticula is lined by columnar epithelium, showing ulceration and active inflammation (b) (diverticulitis). The submucosa also shows mild fibrosis and chronic inflammatory infiltrates (c). Hematoxylin and eosin stain, 2x.

A preoperative abdominal CT scan was suggestive of AA. The postoperative review showed a small diverticulum close to the tip of an inflamed appendix (Figure 7). The patient was admitted and underwent open appendectomy; the procedure was done without any complications. Postoperatively, the patient was doing well without any active complaint; therefore, she was discharged the next day in good health. A review of her histopathological report showed AD with luminal suppuration with no evidence of malignancy (Figure 8). The patient was seen in our clinic for a regular follow-up two weeks postoperatively, and she was doing fine with no active issues. The result of the histopathological report was explained to her.

Case 5

A 44-year-old female, medically and surgically free, presented to the ED with a one-day history of RLQ abdominal pain that started abruptly in association with nausea. This was a first experience, with no previous similar attacks. She had no history of vomiting, fever, or change in bowel motion, and the systemic review was unremarkable. Similarly, the patient denied any history of previous hospital admissions or trauma, and she was not on any medications. Upon physical assessment, she was in pain, afebrile, and hemodynamically stable. Abdominal examination revealed a soft, non-distended abdomen with tenderness in the right inferior fossa and positive rebound tenderness. AA signs were negative, and the WBC count was within normal limits (7.73 × 109/L) (reference range: 4.5-11.0 x 109/L).

**Figure 9:**
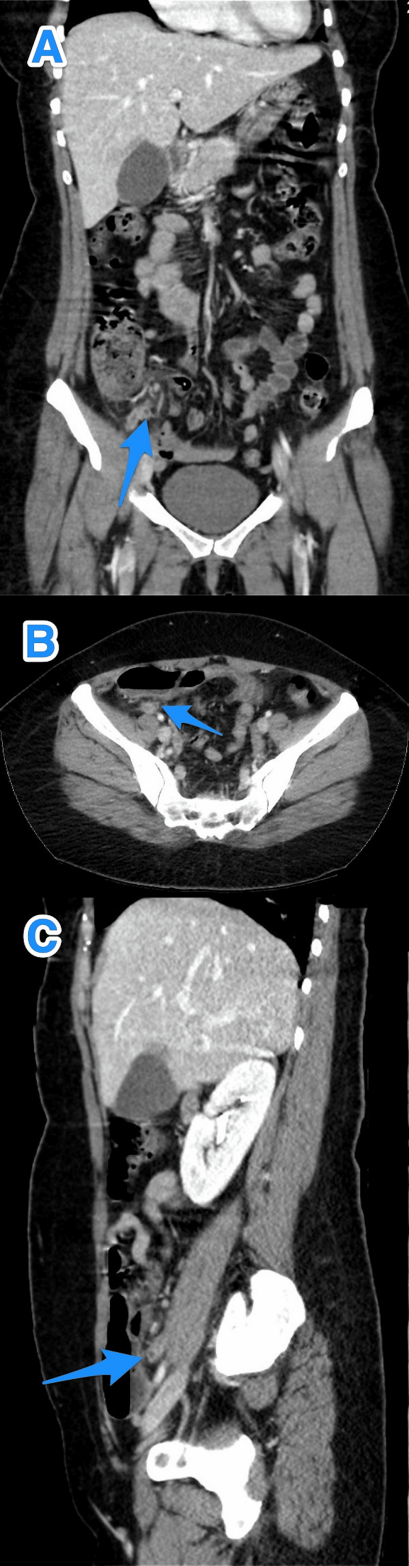
A multiview computed tomography abdomen scan (A-C) shows a small diverticulum close to the tip of an inflamed appendix (arrows).

**Figure 10:**
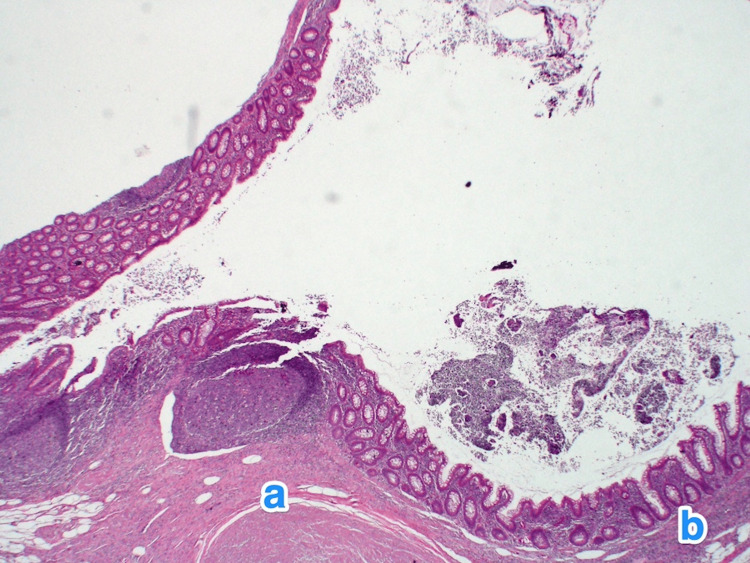
Histopathology of the appendix. A light microscopy photograph of the appendix shows single outpouchings of the mucosa and submucosa through the muscular layer (a) of the appendicular lateral side into the subserosal space. The mucosa of the diverticula is lined by columnar epithelium. The submucosa also shows mild fibrosis and chronic inflammatory infiltrates (b). Hematoxylin and eosin stain, 2x.

A preoperative abdominal CT scan showed a picture consistent with an uncomplicated early AA; however, postoperative assessment showed a small diverticulum close to the tip of an inflamed appendix (Figure 9). She was admitted to our department and underwent a laparoscopic appendectomy on the next day of admission. The procedure was done successfully with no complications. The specimen revealed an inflamed appendix with no diverticula or perforation. Postoperatively, the patient was doing well with no complaints; therefore, she was discharged on the next postoperative day. The histopathological report revealed appendiceal diverticulum with AA and peri-appendicitis (Figure 10). After two weeks, the patient was seen in our clinic and was doing well with no complaints.

## Discussion

AD is a rare condition characterized by the inflammation of the arising diverticulum from the appendix [11]. The progression from diverticulosis to diverticulitis might occur as a result of a partial or complete obstruction of the appendix [12]. Histopathologically, AD is classified as congenital (true diverticula), which includes all the layers of the appendiceal wall (mucosa, submucosa, muscularis layer, and serosa), or acquired (false diverticula), which includes all the layers except the muscular layer [2]. Clinically, most cases are acquired, and the pathogenesis remains unknown [13]. AD is usually overlooked due to its rarity and is usually diagnosed mistakenly with other acute abdomen diseases, such as AA [7-9,13]. We extensively reviewed the English literature and conducted the search in the titles, keywords, and/or abstracts of the articles that were indexed in PubMed using the following keywords: “AD,” “AD mimicking acute appendicitis,” and “appendiceal diverticulosis.” Our findings showed limited published cases in the literature worldwide (approximately 155 cases), few cases in the Arab Gulf region, and only three case reports with no case series in our country [4-7]. In our series, we found five cases, and the findings are summarized in Table 1. The first case was diagnosed as AD during intraoperative evaluation, while the diagnosis of the other four cases was by histopathological evaluation.

**Table 1:** A summary of the five cases of diverticular disease of the appendix. PMHx.: past medical history; PSHX.: past surgical history; Dx: diagnosis; M: male; F: female; N: number; NA: not applicable; F/U: follow-up; M: months; OA: open appendectomy; LA: laparoscopic appendectomy; CT: computed tomography scan of the abdomen; AA: acute appendicitis; WBC: white blood cell count (4-11x10^9/L).

N	Age/sex	PMHx./PSHx.	Symptoms and duration	Examination (+v only)	WBC count	Radiology (diverticula preoperatively?)	Radiology (Dx)	Management	Location of diverticula (CT)	Dx. of diverticulosis by	F/U (M.)	Complications/neoplasms
1	32/F	Gynecological history	RLQ pain for 2 days, associated with nausea, vomiting, anorexia, and fever	RLQ tenderness, positive rebound tenderness	9.93	CT; no	CT; AA	LA	Mid to base	Histopathology	2	-
2	21/M	Unremarkable	RLQ pain for 3 days associated with anorexia	RLQ tenderness	6.35	CT; no	CT; AA	OA	Mid to base	Histopathology	7	-
3	25/M	Unremarkable	RLQ for 4 days associated with nausea and vomiting	RLQ tenderness, positive rebound tenderness	9.8	CT; no	CT; AA	OA	Mid to base	Histopathology	1	-
4	26/F	Unremarkable	RLQ pain for 2 weeks	Mild RLQ tenderness	6.1	CT; no	CT; AA	OA	Mid to base	Histopathology	1.5	-
5	44/F	Unremarkable	RLQ pain for 1 day associated with nausea	RLQ tenderness, positive rebound tenderness	7.73	CT; no	CT; AA	LA	Mid to base	Histopathology	1	-

Recently published articles highlighted the importance of preoperatively establishing the diagnosis due to its strong association with developing multiple complications [14]. Appendiceal perforation is one of its serious complications, specifically for the acquired type due to its thin wall and lack of muscular layer; moreover, the risk of perforation was found to be increased by fourfold compared to AA with a rate of 30-70% [6,15]. In addition to perforation, it has a risk for pseudomyxoma peritonei, gastrointestinal hemorrhage, pelvic pseudocyst formation, and the formation of appendicovesical fistula [15]. Furthermore, a recently published study suggested a prophylactic appendectomy if the AD was found incidentally on radiological investigations due to its strong association with malignancy, mainly carcinoid tumors and mucinous adenomas [14,16]. Hence, it is crucial to differentiate it from other ileocecal diseases and establish the diagnosis preoperatively by clinical and radiological evaluation to intervene in early stages and reduce the risk of developing appendiceal perforation and other associated severe complications [17].

Different clinical features distinguish AD from AA; for instance, the duration of the symptoms is usually longer, ranging from days to years, whereas in AA, the duration is shorter, and the presentation is usually within 48 hours. AD tends to affect the older age group (more than 30 years) with a previous history of similar episodes; in addition, the pain characteristic is intermittent and chronic, while it is acute and persistent in AA [7,9]. Nevertheless, it is challenging to establish the diagnosis by relying on the clinical assessment [18]. Intraoperative AD can be detected if it is large, while the CT scan is considered the modality of choice; however, the diagnosis can be missed if the size is small [10,14,19]. In our series, we performed a retrograde review of the CT scan for all five cases, revealing the presence of the diverticula in all the cases. Histopathological evaluation is the best choice for the definitive diagnosis [7]. However, it is essential to highlight that imaging studies should be considered to diagnose AD preoperatively to initiate treatment in the early stages and reduce morbidity and mortality.

## Conclusions

AD might be an overlooked entity and clinically challenging to distinguish from AA. The majority of AD cases are discovered incidentally during the histopathological examination and retrospective review of CT scans. Since there is a correlation between diverticular disease of the appendix and malignancy, pre- and post-appendectomy CT scans and appendix specimens should be thoroughly analyzed to detect the disease.
